# Supplementary material for: High-dimensional analysis of 16 SARS-CoV-2 vaccine combinations reveals lymphocyte signatures correlating with immunogenicity
Source: Nat Immunol. 2023 Apr 24;24(6):941–54. doi: 10.1038/s41590-023-01499-w (PMC10232362; doi:10.1038/s41590-023-01499-w)
Supplement: Supplementary file 1 — Reporting Summary [file 41590_2023_1499_MOESM1_ESM.pdf]

nature portfolio

Corresponding author(s): Nicolás Nuñez, Mariana Maccioni, Burkhard Becher

Last updated by author(s): 2023/03/14

Reporting Summary

Nature Portfolio wishes to improve the reproducibility of the work that we publish. This form provides structure for consistency and transparency in reporting. For further information on Nature Portfolio policies, see our Editorial Policies and the Editorial Policy Checklist.

Statistics

For all statistical analyses, confirm that the following items are present in the figure legend, table legend, main text, or Methods section.

n/a	Confirmed	
☐	☒	The exact sample size (*n*) for each experimental group/condition, given as a discrete number and unit of measurement
☐	☒	A statement on whether measurements were taken from distinct samples or whether the same sample was measured repeatedly
☐	☒	The statistical test(s) used AND whether they are one- or two-sided<br>*Only common tests should be described solely by name; describe more complex techniques in the Methods section.*
☒	☐	A description of all covariates tested
☐	☒	A description of any assumptions or corrections, such as tests of normality and adjustment for multiple comparisons
☐	☒	A full description of the statistical parameters including central tendency (e.g. means) or other basic estimates (e.g. regression coefficient) AND variation (e.g. standard deviation) or associated estimates of uncertainty (e.g. confidence intervals)
☐	☒	For null hypothesis testing, the test statistic (e.g. *F*, *t*, *r*) with confidence intervals, effect sizes, degrees of freedom and *P* value noted<br>*Give P values as exact values whenever suitable.*
☒	☐	For Bayesian analysis, information on the choice of priors and Markov chain Monte Carlo settings
☒	☐	For hierarchical and complex designs, identification of the appropriate level for tests and full reporting of outcomes
☒	☐	Estimates of effect sizes (e.g. Cohen's *d*, Pearson's *r*), indicating how they were calculated

Our web collection on statistics for biologists contains articles on many of the points above.

Software and code

Policy information about availability of computer code

Data collection	SpectroFlo Software - Cytek Aurora (Cytek)<br>AID ELISpot software version 7.0 (AID Autoimmun Diagnostika GmbH) - AID Classic ELISpot Reader
Data analysis	FlowJo Software (10.6.1, FlowJo LLC, BD Life Sciences), Affinity designer,<br>R version 4.0.1 (R Core Team 2020):<br>R studio 1.3.959, dplyr, FlowSOM, flowStats, ggplot2, Hmisc, pheatmap, Stats, UMAP, flowcore

For manuscripts utilizing custom algorithms or software that are central to the research but not yet described in published literature, software must be made available to editors and reviewers. We strongly encourage code deposition in a community repository (e.g. GitHub). See the Nature Portfolio guidelines for submitting code & software for further information.

Data

Policy information about availability of data

All manuscripts must include a data availability statement. This statement should provide the following information, where applicable:

- Accession codes, unique identifiers, or web links for publicly available datasets
- A description of any restrictions on data availability
- For clinical datasets or third party data, please ensure that the statement adheres to our policy

This study did not generate new reagents. Data are available in a public repository (https://doi.org/10.5281/zenodo.7734088). This study did not generate new codes. The codes that support these findings have been previously described54–63.

Human research participants

Policy information about studies involving human research participants and Sex and Gender in Research.

Reporting on sex and gender	Data relating to the gender was collected on the open Phase IIB clinical trial - ECEHeVac, NCT04988048
Population characteristics	497 volunteers (age range 18-82 years old) were enrolled in a randomized, open Phase IIB clinical trial (ECEHeVac, NCT04988048) aimed at comparing the immunogenicity and reactogenicity of heterologous and homologous vaccination regimens available in Córdoba, Argentina.
Recruitment	Eligible participants were healthy volunteers older than 18 years who had received a first dose of the AZD, BBIBP, Sput 26, or mRNA-1273 vaccine 30-120 days prior to the enrolment date. Exclusion criteria were: immunocompromised status with underlying disease or immunosuppressive treatment; pregnancy and lactation; having received a major surgical intervention in the 30 days prior to the enrolment date; having had a severe allergic reaction (anaphylaxis) to any vaccine; having a visceral disease that lead to disability (heart failure, kidney failure, respiratory failure, liver failure, intestinal malformations, electro-dependence, or having had a visceral transplant less than 2 years previously); and having had COVID-19 (symptomatic or asymptomatic) or a positive anti-nucleocapsid IgG via ELISA on T1 (except for those subjects that had been vaccinated with BBIBP as the first dose).<br>Randomization was performed centrally at the Epidemiology Area of the Ministry of Health of the Province of Córdoba by assigning codes to the participants at the time of their registration, anonymizing their personal information to avoid possible biases.<br>Randomization methodology: A list was prepared with participants who met inclusion criteria and did not present exclusion criteria. Randomization was performed with a equal group allocation using random permuted block stratification. Randomization was stratified by age for the groups of 18 to 59 years or 60 and over, and according to the time since the application of the first dose of vaccine (0 to 30 days and 30 to 60 days).
Ethics oversight	The study received ethical approval by the Registro Provincial de Investigación en Salud (Provincial Registry of Health Research, REPIS-Cba #4371)

Note that full information on the approval of the study protocol must also be provided in the manuscript.

Field-specific reporting

Please select the one below that is the best fit for your research. If you are not sure, read the appropriate sections before making your selection.

☒ Life sciences ☐ Behavioural & social sciences ☐ Ecological, evolutionary & environmental sciences

For a reference copy of the document with all sections, see nature.com/documents/nr-reporting-summary-flat.pdf

Life sciences study design

All studies must disclose on these points even when the disclosure is negative.

Sample size	No sample size calculation was performed. We used all the samples available in each dataset.
Data exclusions	For FACS, samples with fewer than 1000 live cells were excluded. For ELISpot, samples were excluded if the negative control wells had more than 39 or the positive control wells fewer than 40 spots.
Replication	All experiments have multiple replicates. All results were performed in at least 2 independent experiments.
Randomization	Participants were randomized with equal group allocation to determine the vaccine used as Dose 2
Blinding	All investigators and collaborators were blinded to clinical results when performing measurements and assays.

Reporting for specific materials, systems and methods

We require information from authors about some types of materials, experimental systems and methods used in many studies. Here, indicate whether each material, system or method listed is relevant to your study. If you are not sure if a list item applies to your research, read the appropriate section before selecting a response.

Materials & experimental systems

n/a	Involved in the study
☐	☒ Antibodies
☐	☒ Eukaryotic cell lines
☒	☐ Palaeontology and archaeology
☒	☐ Animals and other organisms
☒	☐ Clinical data
☒	☐ Dual use research of concern

Methods

n/a	Involved in the study
☒	☐ ChIP-seq
☐	☒ Flow cytometry
☒	☐ MRI-based neuroimaging

Antibodies

Antibodies used	The antibodies are listed in Extended data table 2 and Extended data table 3.
Validation	All the antibodies have been validated by the manufacturer and then titrated in house (human PBMCs). Please see clones, fluorochromes and company webpages for specific validation.

Eukaryotic cell lines

Policy information about cell lines and Sex and Gender in Research

Cell line source(s)	Vero 76 cells (ATCC CRL-1587)
Authentication	No authentication for the commercially available cell line.
Mycoplasma contamination	Vero 76 cell line was tested negative for mycoplasma contamination.
Commonly misidentified lines (See ICLAC register)	No commonly misidentified cell lines were used

Flow Cytometry

Plots

Confirm that:

☒ The axis labels state the marker and fluorochrome used (e.g. CD4-FITC).

☒ The axis scales are clearly visible. Include numbers along axes only for bottom left plot of group (a 'group' is an analysis of identical markers).

☒ All plots are contour plots with outliers or pseudocolor plots.

☒ A numerical value for number of cells or percentage (with statistics) is provided.

Methodology

Sample preparation	Cryopreserved PBMCs were stored in liquid nitrogen. Then, for spectral flow analysis and ELISpot, cells were thawed using Cryo thaw devices (Medax). PBMCs were resuspended in cell culture medium supplemented with 2U/ml benzonase by centrifugation (300 r.c.f.; 7 min; 24C). Cell count was calculated using an automated cell counter (Bio-Rad).For the spike-binding mBC and T cell panels, 1.5x106 and 1.0x106 PBMCs respectively were washed with PBS and blocked using Human TruStain FcX and True-Stain Monocyte Blocker (BioLegend).
Instrument	Cytek Aurora (Cytek)
Software	SpectroFlo Software
Cell population abundance	Expressed as a frequency of the selected population

Gating strategy	Gating strategy is provided in Extended data 7. For high-dimensional flow cytometry analysis, dead cells, doublets, or cells stained by fluorochrome aggregates were excluded from the analysis via manual gating using FlowJo. Datasets of different batches were corrected using the CytoNorm R package

☒ Tick this box to confirm that a figure exemplifying the gating strategy is provided in the Supplementary Information.
