# Supplementary material for: High-dimensional analysis of 16 SARS-CoV-2 vaccine combinations reveals lymphocyte signatures correlating with immunogenicity
Source: Nat Immunol. 2023 Apr 24;24(6):941–54. doi: 10.1038/s41590-023-01499-w (PMC10232362; doi:10.1038/s41590-023-01499-w)
Supplement: Supplementary file 4 — Flow cytometry antibodies (B cell panel). [file 41590_2023_1499_MOESM4_ESM.pdf]

Flow cytometry antibodies – B cell panel

Surface				
**Fluorochrome**	**Antigen**	**Clone**	**Dilution**	**Company**
BV785	CCR7	G043H7	1/100	Biolegend
BV650	CXCR3	G025H7	1/25	Biolegend
BUV615-p	CCR4	1G1	1/100	BD
BV711	CCR6	G034E3	1/40	Biolegend
BV750	CXCR5	RF8B2	1/40	BD
PE	CXCR4	QA18A64	1/150	Biolegend
BUV395	CD45RA	HI100	1/1500	BD
Pacific Blue	CD62L	DREG-56	1/200	
BUV496	CD16	3G8	1/100	BD
BUV805	CD3	UCHT1	1/50	BD
BV570	CD20	2H7	1/40	Biolegend
Pe-Cy5.5	CD19	HIB19	1/250	LifeTechnologies
BUV737	CD56	NCAM16.2	1/100	BD
PE/Dazzle™ 594	IgM	MHM-88	1/650	Biolegend
BUV563	CD27	M-T271	1/100	BD
Spark NIR	CD14	63D3	1/200	Biolegend
Spark Blue 550	CD4	SK3	1/250	Biolegend
BV605	PD1	EH12.2H7	1/40	Biolegend
AF647	IgG	Polyclonal	1/5000	Jackson
PerCP	CD45	2D1	1/75	Biolegend
BB700	BAFFR	11C1	1/200	BD
BV510	CD127	A019D5	1/50	Biolegend
APC-Fire 810	CD38	HIT2	1/150	Biolegend
Pe-Cy7	CD25	M-A251	1/50	Biolegend
APC Fire 750	CD21	Bu32	1/50	Biolegend
R718	CD8	SK1	1/500	BD
FITC	IgA	Polyclonal	1/1000	Jackson
BV480	IgD	B56	1/150	BD
