# Supplementary material for: High-dimensional analysis of 16 SARS-CoV-2 vaccine combinations reveals lymphocyte signatures correlating with immunogenicity
Source: Nat Immunol. 2023 Apr 24;24(6):941–54. doi: 10.1038/s41590-023-01499-w (PMC10232362; doi:10.1038/s41590-023-01499-w)
Supplement: Supplementary file 5 — Flow cytometry antibodies (T cell panel). [file 41590_2023_1499_MOESM5_ESM.pdf]

Flow cytometry antibodies - T cell panel

Surface				
**Fluorochrome**	**Antigen**	**Clone**	**Dilution**	**Company**
BV785	CCR7	G043H7	1/100	Biolegend
BV650	CXCR3	G025H7	1/25	Biolegend
BUV615-p	CCR4	1G1	1/100	BD
BUV395	TIGIT	747845	1/50	BD
BUV661	HLA-DR	G46-6	1/300	BD
BUV805	CD3	UCHT1	1/75	BD
BV570	CD8	RPA-T8	1/150	Biolegend
Pe-Cy5.5	CD45RA	MEM-56	1/125	LifeTechnologies
BUV737	CD16	3G8	1/300	BD
PE/Dazzle™ 594	CD56	5.1H11	1/150	Biolegend
PE-Cy5	TCR PANgd	IMMU510	1/75	Beckman Coulter
BV421	CD69	FN50	1/100	Biolegend
Biotin	CD103	Ber-ACT8	1/140	Biolegend
BUV563	CD27	M-T271	1/100	BD
Spark NIR	CD14	63D3	1/400	Biolegend
Spark Blue 550	CD4	SK3	1/100	Biolegend
BV605	PD1	EH12.2H7	1/35	Biolegend
BB660	CD1c	F10/21A3	1/200	BD
PerCP-eFluor 710	CD39	A1	1/75	ThermoScientific
APC	CD95	GB11	1/150	BD
Super Bright 436	CD123	6H6	1/100	ThermoScientific
PE-Fire810	KLRG1	SA231A2	1/150	Biolegend
PerCP	CD45	2D1	1/100	Biolegend
PE	TCR Vα7.2	3C10	1/130	Biolegend
BB700	TIM3	344823	1/75	BD
BV510	CD127	A019D5	1/50	Biolegend
BV750	CD19	SJ25C1	1/150	BD
APC-Fire 810	CD38	HIT2	1/100	Biolegend
**Intracellular**				
**Fluorochrome**	**Antigen**	**Clone**	**Isotype**	**Company**
Pe-Cy7	FOXP3	236A/E7	1/25	ThermoScientific
APC-eF780	EOMES	WD1928	1/30	ThermoScientific
BB790-P	CD152 (CTLA-4)	BNI3	1/50	BD
BV711	Tbet	4B10	1/50	Biolegend
AF700	GZMB	GB11	1/250	BD
AF488	TCF1	C63D9	1/400	Cell Signaling
BV480	Ki-67	B56	1/130	BD
BB630	Streptavidin		1/500	BD
